# Highly Dynamic Host Actin Reorganization around Developing *Plasmodium* Inside Hepatocytes

**DOI:** 10.1371/journal.pone.0029408

**Published:** 2012-01-06

**Authors:** Carina S. S. Gomes-Santos, Maurice A. Itoe, Cristina Afonso, Ricardo Henriques, Rui Gardner, Nuno Sepúlveda, Pedro D. Simões, Helena Raquel, António Paulo Almeida, Luis F. Moita, Friedrich Frischknecht, Maria M. Mota

**Affiliations:** 1 Malaria Unit, Instituto de Medicina Molecular, Faculdade de Medicina Universidade de Lisboa, Lisboa, Portugal; 2 PhD Programme in Experimental Biology and Biomedicine, Center for Neuroscience and Cell Biology, University of Coimbra, Coimbra, Portugal; 3 Cell Biology Unit, Instituto de Medicina Molecular, Universidade de Lisboa, Lisboa, Portugal; 4 Instituto Gulbenkian de Ciência, Oeiras, Portugal; 5 Center of Statistics and Applications, University of Lisbon, Lisboa, Portugal; 6 Cell Biology of the Immune System Unit, Instituto de Medicina Molecular, Universidade de Lisboa, Lisboa, Portugal; 7 Unidade de Entomologia Médica/UPMM, Instituto de Higiene e Medicina Tropical, Universidade Nova de Lisboa, Lisboa, Portugal; 8 Parasitology, Department of Infectious Diseases, University of Heidelberg Medical School, University of Heidelberg, Heidelberg, Germany; Seattle Biomedical Research Institute - University of Washington, United States of America

## Abstract

*Plasmodium* sporozoites are transmitted by *Anopheles* mosquitoes and infect hepatocytes, where a single sporozoite replicates into thousands of merozoites inside a parasitophorous vacuole. The nature of the *Plasmodium*-host cell interface, as well as the interactions occurring between these two organisms, remains largely unknown. Here we show that highly dynamic hepatocyte actin reorganization events occur around developing *Plasmodium berghei* parasites inside human hepatoma cells. Actin reorganization is most prominent between 10 to 16 hours post infection and depends on the actin severing and capping protein, gelsolin. Live cell imaging studies also suggest that the hepatocyte cytoskeleton may contribute to parasite elimination during *Plasmodium* development in the liver.

## Introduction

Diverse pathogens have developed numerous strategies to successfully survive and replicate inside host cells. They often subvert signalling pathways and cytoskeletal components of the host cell to avoid the host's immune system and for direct access to host metabolites [1]. The bacteria *Salmonella enterica*, for example, induces host cell actin reorganization and ruffle formation to facilitate its internalization into non-phagocytic cells [2], while the vaccinia virus moves along microtubules and polymerizes actin to form comet tails that allow viral dissemination into non-infected cells [reviewed in 3]. Besides bacteria and viruses, protozoan parasites are also capable of manipulating the host cell cytoskeleton. The apicomplexan parasite *Cryptosporidium parvum*, responsible for diarrheal illness, induces the reorganization of the host's actin network into a plaque-like structure that separates the parasite from the cell cytoplasm, thereby creating an intracellular but extracytoplasmic niche, where it replicates [4]. During *Theileria* infection, host microtubules associate with the parasite, which divides in synchrony with the host cell [5], [6]. Invasion by *Toxoplasma gondii* induces the formation of a host F-actin ring at the junction site [7] and, at a later stage of infection, *T. gondii* recruits microtubules, proposed to form conduits along which host organelles are transported to the parasitophorous vacuole [8].

The malaria parasites (*Plasmodium* spp.) of mammals first replicate asexually in hepatocytes and later in red blood cells (RBCs). Several studies show that, within RBCs, *Plasmodium* exports proteins to the host cell cytosol that manipulate the host cell cytoskeleton, important for parasite egress and progression of infection [9], [10], [11]. However, during the liver stage of infection, few reports exist on the interaction of the host cell cytoskeleton with *Plasmodium*. While no significant reorganization of host microtubules or actin was reported in fixed cells at 24 hours of *Plasmodium* spp. development [12], an F-actin ring in the cell–parasite junction was observed during invasion of hepatocytes by sporozoites [7]. Here, we investigate the hepatocyte actin and microtubule organization during *Plasmodium berghei* development, using live cell imaging.

## Results and Discussion

### Reorganization of hepatocyte actin, but not tubulin, occurs around developing *P. berghei*

To investigate a potential reorganization of the hepatocyte cytoskeleton during *Plasmodium* infection, we established Huh7 cell lines stably expressing mCherry::β-actin or mCherry::α-tubulin fusion proteins. Anti-α-tubulin antibody or phalloidin labelling showed that all microtubules stained with the antibody were positive for mCherry::α-tubulin and all the filamentous actin (F-actin) structures stained with phalloidin, were also positive for mCherry::β-actin, showing integration of exogenous proteins into the microtubules or the F-actin of the living cells, respectively (Fig. S1). Transformed cell lines were indistinguishable from the parent lines, with unperturbed key cellular events involving cytoskeletal dynamics in both mCherry lines (data not shown). Furthermore, infection of these cells with GFP-Pb proceeded at the same rate as in control Huh7 cells (Fig. S2).

**Figure 1:**
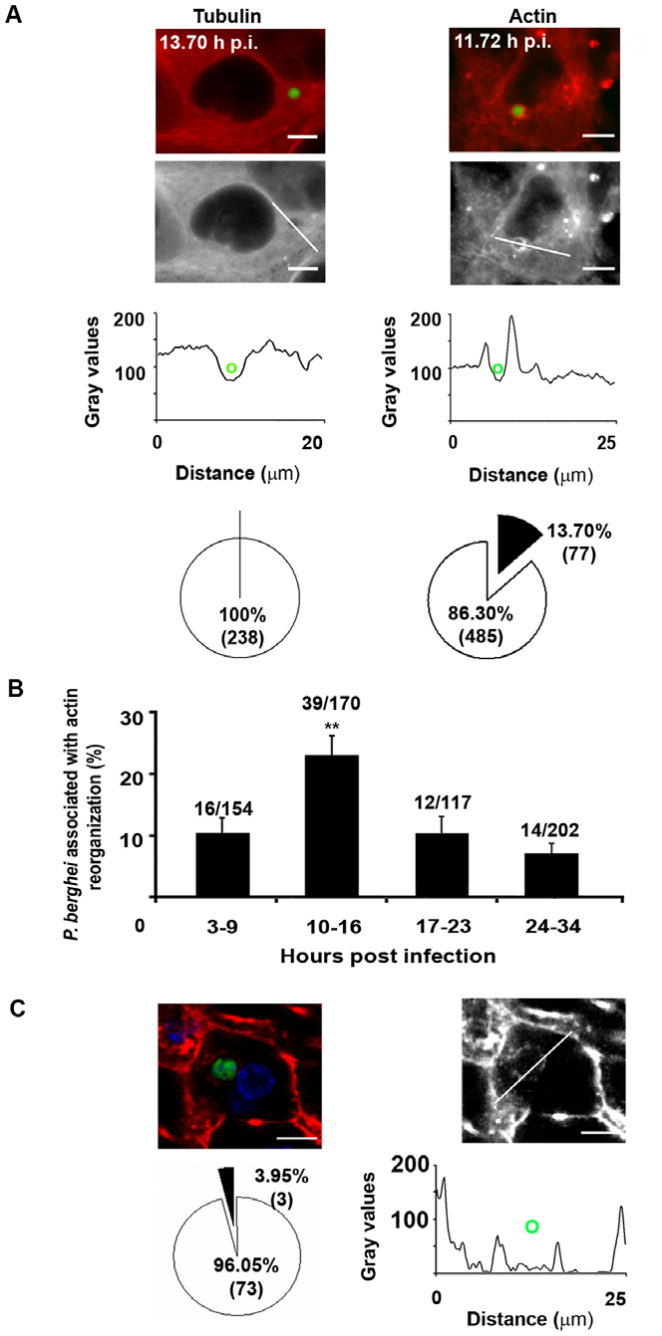
Hepatocyte cytoskeleton reorganization around developing P. berghei *in vitro* and *in vivo*. (**A**) Actin but not tubulin is reorganized in Huh7 cell lines infected with GFP-Pb parasites (red and grey: mCherry::α-tubulin or mCherry::β-actin; green: GFP-Pb). Plot profiles represent the pixel intensity (gray values) of the regions indicated in the pictures (lines); **o** represents the parasite. Pie plots represent the percentages and absolute numbers of parasites associated (▪) or not (□) with host cytoskeleton reorganization. (**B**) Distribution of the percentage of *P. berghei* parasites associated with host cell actin reorganization in different infection periods. Numbers of parasites associated with actin reorganization/total numbers of parasites analysed are indicated above the bars. (**C**) Hepatocyte F-actin around *P. berghei* EEFs at 24 hours p.i. in BALB/c mice livers (red and grey: Phalloidin AlexaFluor 594; green: GFP-Pb; blue: nuclei). The pie plot represents the percentage and absolute numbers of parasites associated (▪) or not (□) with host actin reorganization. Plot profile represents the pixel intensity (gray values) along the white line; **o** represents the parasite. Error bars represent Standard Errors ** p<0.01, scale bars represent 10 µm.

We next aimed to identify the possible interactions between these components of the host cell cytoskeleton and the developing *Plasmodium* parasite. Cells from both cell lines were infected with GFP-*Plasmodium berghei* (GFP-Pb) sporozoites and observed by wide field fluorescence microscopy at different times after infection. Time lapse experiments, with 20 seconds acquisition intervals, were performed between 3 and 34 hours p.i. to follow the host cytoskeleton dynamics around the parasite during its development. No significant host microtubule reorganization was observed around 238 GFP-Pb parasites (Fig. 1A; Movie S1). However, clear host cell actin reorganization events, characterized by changes of mCherry::β-actin fluorescence around the parasites, were observed around 77 out of 562 developing GFP-Pb (14±2%) analysed between 3 and 34 hours p.i. (Fig. 1A; Movie S2). Host actin reorganization events were highly dynamic, comprising cycles of polymerization and depolymerization around the parasite (Movie S2). Data analysis showed that, although present throughout infection, this phenomenon occurred preferentially between 10 to 16 hours p.i. (23±3%, p<0.01) (Fig. 1B). Although not much is known about the biological processes occurring during intra-hepatic *Plasmodium* development, the interval between 10 and 16 hours p.i. may coincide with an important step in the preparation for the extensive nuclear replication that starts soon after that period [13]. We next determined whether actin polymerization around developing GFP-Pb also occurs during liver infection *in vivo*, by staining liver slices of BALB/c mice 24 hours p.i., with the F-actin binding toxin fluorescent conjugated, phalloidin. Clear actin rings were observed around approximately 4% (3 out of 76) of the exoerythrocytic forms (EEFs) analysed by fluorescence confocal microscopy (Fig. 1C). Thus, our observations show that hepatocyte actin reorganization events also occur during EEF development in the liver of infected mice. The lower percentage of actin rings around mouse liver EEFs, compared to the live cell imaging experiments formerly presented, is also observed *in vitro* around fixed EEFs (data not shown). This is probably due to the fact that the observed actin polymerization events are extremely dynamic, as shown in our live cell imaging experiments, and as such much more difficult to capture in fixed conditions.

Dynamic actin reorganization is not only associated with certain intracellular microorganisms, such as bacteria and viruses, but also with plasma membrane perturbations or even with inert particles, like beads [14]. Thus, we decided to investigate whether the actin reorganization that was being observed around developing *P. berghei* EEFs was a *Plasmodium*-induced phenomenon, common to apicomplexan parasites or simply occurring in response to the presence of a foreign body. To that end, time lapse experiments were performed with: (i) 3 µm polyamino uncoated beads (corresponding to the size of *P. berghei* parasites between 10 and 16 hours p.i.) internalized by Huh7 mCherry::β-actin cells and (ii) Huh7 mCherry::β-actin cells infected with another apicomplexa parasite, GFP-expressing *T. gondii* tachyzoites. Although Huh7 cells are not professional phagocytes, they clearly internalize beads following a 1 hour starvation period. These experiments were performed between 10 and 16 hours post-bead internalization or *T. gondii* infection, corresponding to the interval of *P. berghei* infection where there is the highest percentage of *Plasmodium* parasites associated with host actin reorganization, employing experimental conditions that mimicked infection by *Plasmodium* (see Material and Methods).

**Figure 2:**
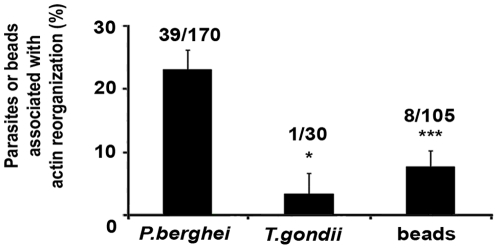
Hepatocyte cytoskeleton reorganization depends on the presence of *P. berghei*. Percentage of parasites (*P. berghei* and *T. gondii*) and beads associated with hepatocyte actin reorganization between 10 and 16 hours p.i. or bead internalization. Numbers of parasites or beads associated with actin reorganization/total numbers of parasites or beads analysed are indicated above the bars. Error bars represent Standard Errors * p<0.05 *** p≤0.001.

Comparison of actin reorganization events, during *P. berghei* infection (22.9±3%), *T. gondii* infection (3.3%, s.e. = 3%, CI95 = (0.1%,17%)) or after internalization of beads (7.6%, s.e. = 3%, CI95 = (3.5%,14.5%)), showed that the events observed around *T. gondii* or beads were significantly less frequent than those occurring around *Plasmodium* (Fig. 2). The few events occurring around beads or *T. gondii* were also less intense and dynamic than those observed around *P. berghei* in the same period of time (Movie S3). Previous work by Yam and colleagues show that similar actin dynamics can be observed around E-cadherin covered beads, *E. coli* and *Listeria*-containing phagosomes in MDCK cells [14]. However, our work shows that, in hepatocytes, the actin dynamics around *Plasmodium* is significantly more frequent and intense, when compared to the one observed around uncoated beads or the related parasite, *T. gondii*. This suggests that, although the phenomena that we observe may be part of the normal actin dynamics of the cells, specific features of the *Plasmodium*, or of its parasitophorous vacuole membrane, also play an important role in the process.

### Dynamics of hepatocyte actin reorganization events associated with developing *P. berghei*

**Figure 3:**
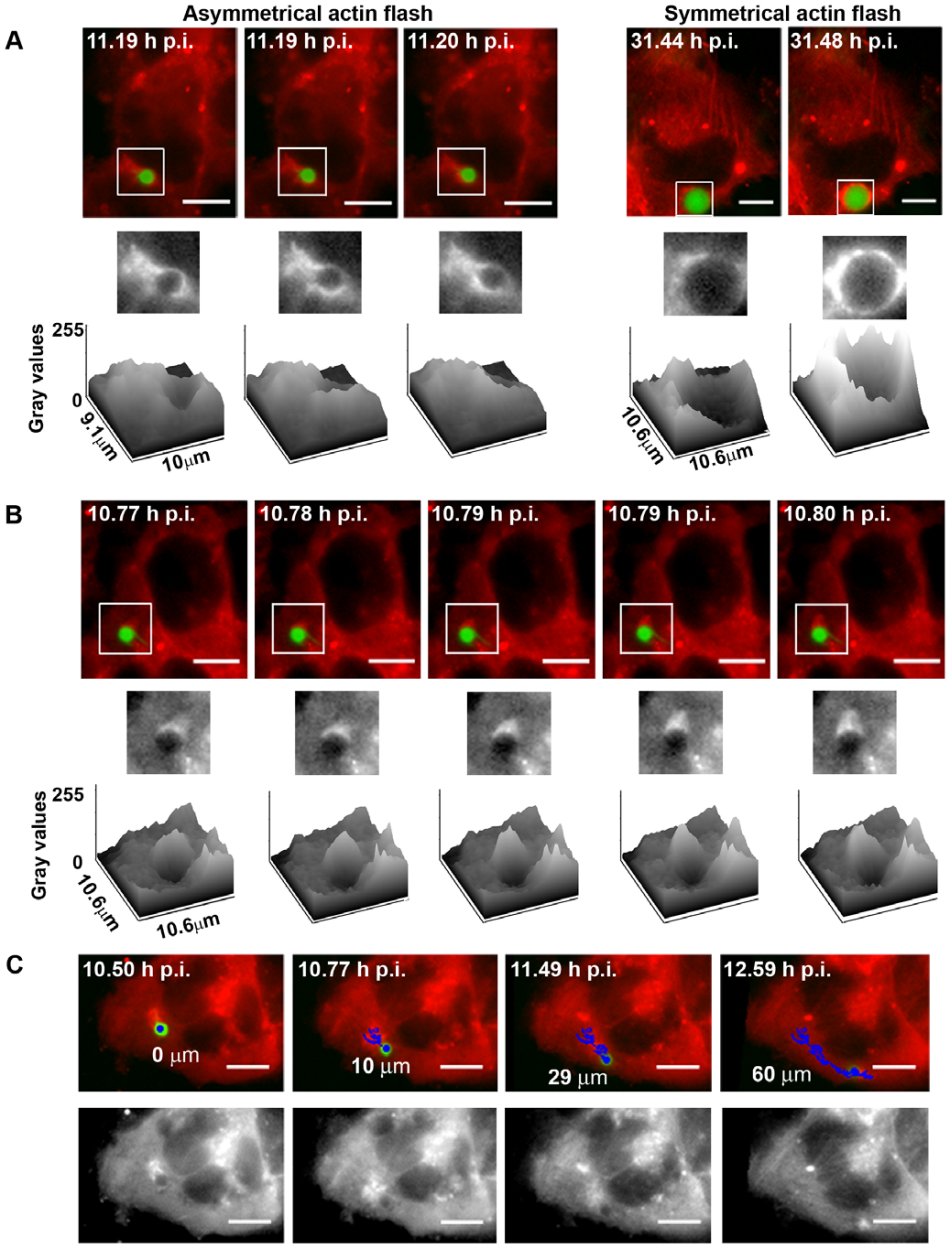
Dynamics of hepatocyte actin clouds around developing *P. berghei*. Asymmetrical and symmetrical actin clouds (**A**) as well as tail-like actin clouds (**B**) are present around GFP-Pb (red and grey: mCherry::β-actin; green: GFP-Pb), surface plots represent the intensity of the pixels of the mCherry::β-actin channel in the selected region (square). (**C**) Translocation of a GFP-Pb, associated with a tail-like actin cloud. The tracked path is indicated in blue (red and grey: mCherry::β-actin; green: GFP-Pb). Scale bars represent 10 µm. Some frames present the same time points as the acquisition interval between individual images was 20 s ( = 0.0056 h) and thus too small to annotate in consecutive frames, when using a time scale of hours.

We termed the actin reorganization events around *Plasmodium* actin clouds. The dynamics of actin clouds were characterized by the accumulation of actin in close vicinity of the PV, which appeared either asymmetrical, moving around the vacuole (Fig. 3A; Movie S4), or symmetrical, surrounding the entire PV (Fig. 3A; Movie S2). Actin clouds could also rearrange into polar structures reminiscent of actin tails (Fig. 3B; Movie S5). Actin reorganization could last for the whole duration of the movie, e.g. 2 hours, while others lasted for just a few minutes or even seconds. In all cases, actin clouds were very dynamic, as seen in Movies S4 and S5.

The parasites associated with actin clouds usually showed a non progressive movement (Movie S4). However, some parasites associated with tail-like actin clouds were clearly prone to translocation inside the cell. The longest distance observed for an intracellular parasite translocation event was 60 µm, which occurred between two positions separated by 19 µm within the cell (Fig. 3C). No specific directional pattern was observed from 12 moving parasites.

Host cell actin reorganization observed around *P. berghei* resembles the type of actin dynamics associated with other pathogenic systems, such as *Listeria*-containing phagosomes [14], or even with normal cellular functions, such as the movement of endosomes or other intracellular vesicles inside the cell cytosol [15], [16]. Similarly, *Plasmodium* is also surrounded by the parasitophorous vacuole membrane, suggesting that actin dynamics observed around this apicomplexan parasite might originate at this membrane. Indeed, it has been demonstrated that membranes can be associated with actin polymerization events, depending on their lipid content [17]. Considering this, it is tempting to hypothesize that the actin dynamics observed around *Plasmodium* might be related with active vacuole membrane remodelling as part of the vacuole maturation process during parasite development.

### Gelsolin is involved in host actin reorganization associated with developing *P. berghei*

**Figure 4:**
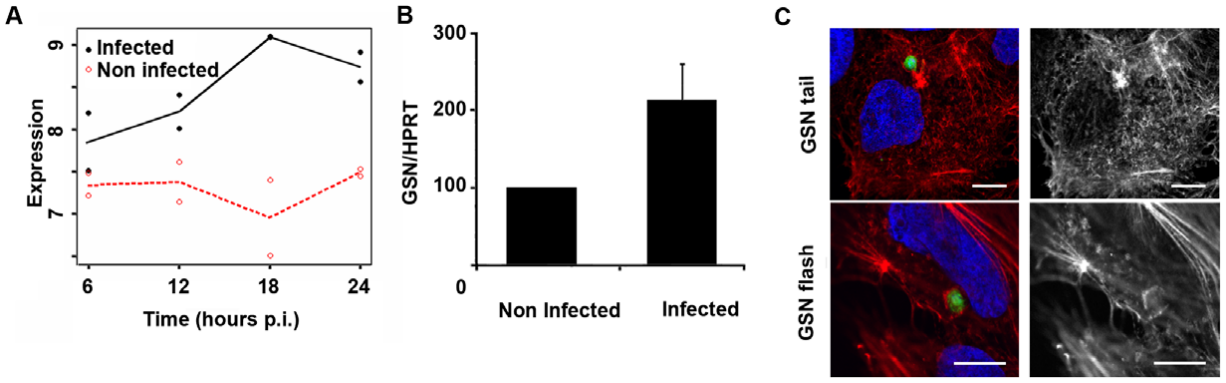
Gelsolin expression and distribution around developing *P. berghei*. (**A**) Microarray data of GSN expression levels in *P. berghei*- infected and non infected Hepa1-6 cells at different time points of infection, from [18] (**B**) qRT-PCR data of GSN expression in *P. berghei* infected and non-infected Huh7 cells at 12 hours p.i.. (**C**) Huh7 cells showing an accumulation of GSN around GFP-Pb in a pattern similar to an actin cloud and tail-like actin cloud (red and grey: anti-GSN antibody, green: GFP-Pb; blue: nuclei). Scale bars represent 10 µm.

We next sought to identify actin related proteins involved in the dynamic events observed around the developing *Plasmodium*. A recent microarray screen of *Plasmodium*-infected versus non-infected cells [18] showed that gelsolin (GSN) transcripts are significantly up-regulated throughout infection, during all time points of infection analysed in that study (Fig. 4A). GSN severs actin filaments and remains attached to the barbed end of the short filament, preventing elongation. When GSN uncaps these filaments, many actin polymerization points become available in the cell cytosol to generate new actin filaments [19]. We confirmed the microarray data by quantitative RT-PCR (qRT-PCR), comparing infected Huh7 cells 12 hours p.i. and non-infected cells (Fig. 4B). When infected Huh7 cells were stained with an anti-GSN antibody, structures resembling actin clouds were observed (Fig. 4C).

**Figure 5:**
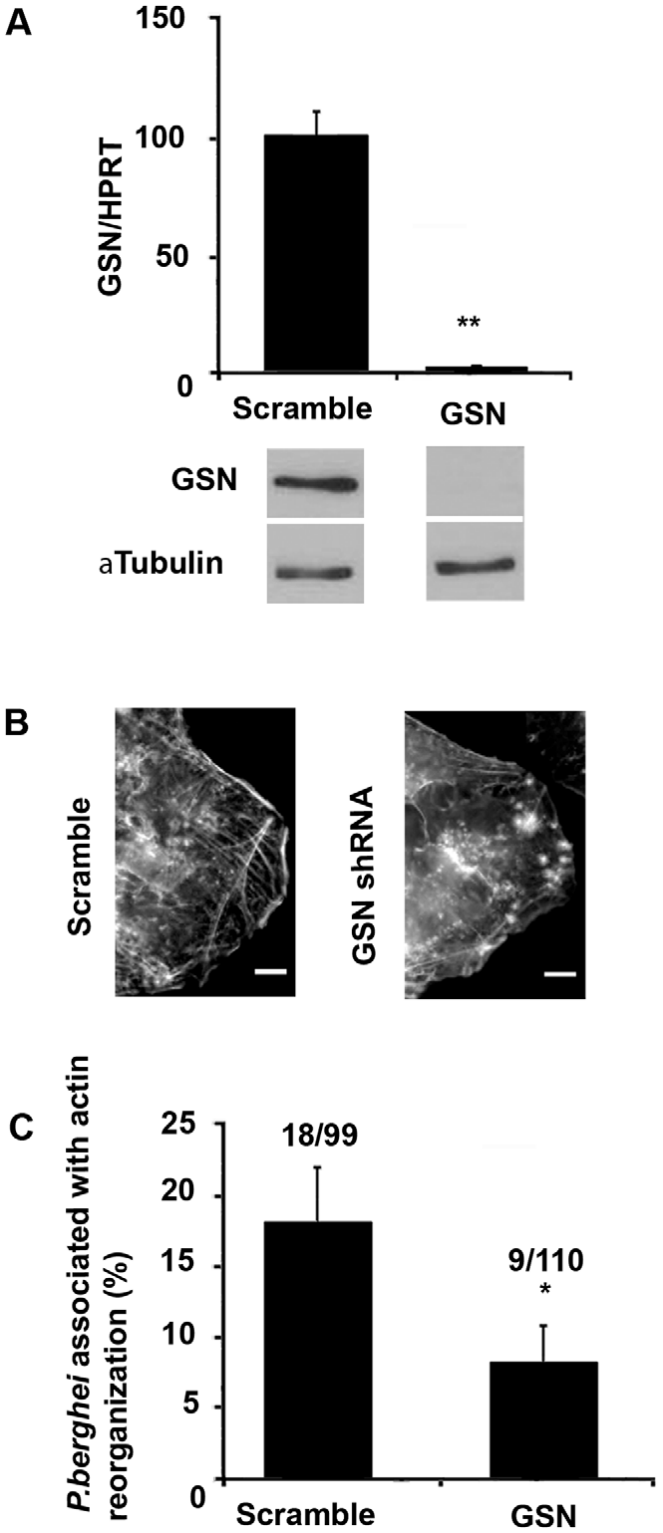
Gelsolin is important for actin reorganization around developing *P. berghei* and parasite disappearance. (**A**) GSN mRNA (top) and protein (bottom) expression after knockdown with shRNA. GSN and α-tubulin bands corresponds to 93 kDa and 55 kDa molecular weights, respectively. (**B**) Phalloidin staining of Huh7 cells transduced with Scramble and GSN shRNA (grey: F-actin). (**C**) Effect of GSN knockdown on the percentage of *P. berghei* parasites associated with hepatocyte actin reorganization between 10 and 16 hours p.i.. Numbers of parasites associated with actin reorganization/total numbers of parasites analysed are indicated above the bars. Error bars represent Standard Errors * p<0.05 ** p<0.01; scale bars represent 10 µm.

To determine the contribution of GSN to actin reorganization events, we performed GSN knockdown experiments in the Huh7 mCherry::β-actin cell line by lentiviral-delivered shRNA (Fig. 5A), followed by infection with GFP-Pb sporozoites. Time lapse experiments of *Plasmodium* infection (10 to 16 hours p.i.) showed that when GSN expression is efficiently down-modulated (97.5±0.5%, Fig. 5A), actin structures are perturbed (Fig. 5B) and the percentage of parasites associated with actin clouds is significantly lower than that observed in the control situation (Fig. 5C). Attempts to deplete other actin related proteins, such as Arp2 and Arp3 from the Arp2/3 complex as well as N-WASP, usually involved in this type of actin phenomenon, were made but without success due to significant cell death after knockdown of these proteins. The results clearly show that, although other proteins are also likely to be involved in *P. berghei*-associated hepatocyte actin reorganization, GSN is an important player in this process. Most probably contributing to the actin turnover and consequently the dynamics of the actin clouds.

### Biological relevance of hepatocyte actin reorganization around developing *Plasmodium*

**Figure 6:**
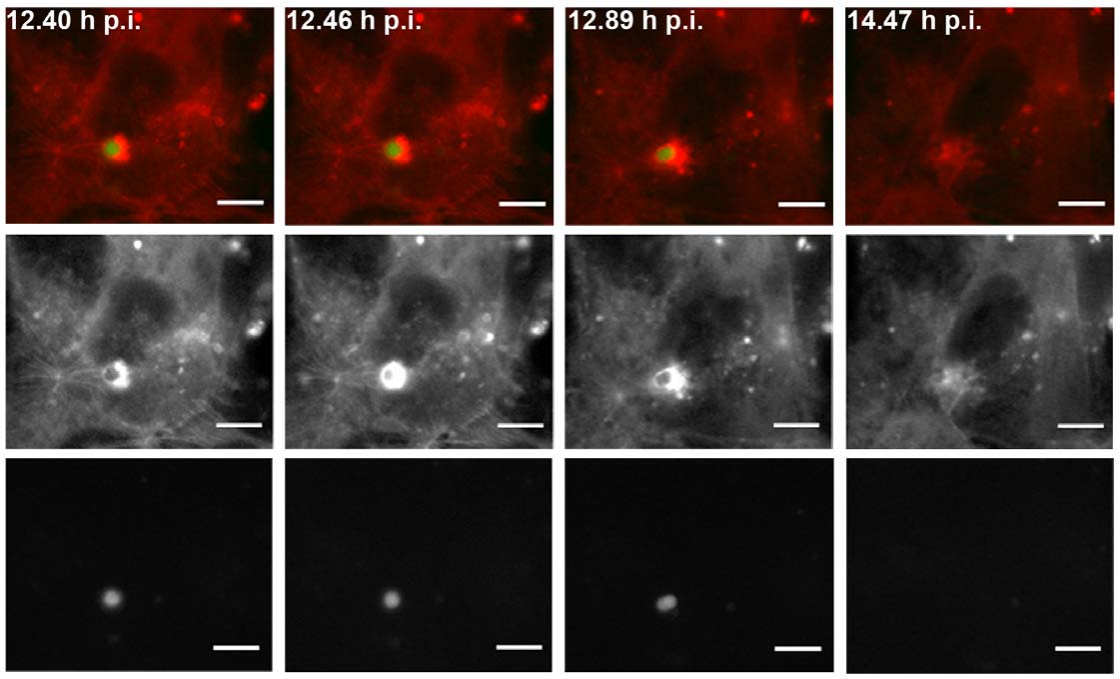
Actin reorganization is associated with *P. berghei* disappearance. Frames of movie S6 where it is possible to observe parasite deformation and disappearance coincident with hepatocyte actin reorganization (red: mCherry::β-actin; green: GFP-Pb; grey pictures represent single channels), scale bars represent 10 µm.

Many pathogens hijack the host cytoskeleton for their own benefit, either to spread from cell to cell or to capture important nutrients [3]. During live cell imaging experiments we noticed the disappearance of some parasites to coincide with extremely dynamic actin events. Indeed, 5.2% of the parasites recorded during actin reorganization events disappear (Fig. 6; Movie S6; Table S1). In the experiment shown in movie S6, a progressively stronger actin cloud around the vacuole was observed, which first deformed and finally eliminated the parasite leading to an apparent vacuole closure (Fig. 6). In contrast, only 0.8% of the parasites not associated with actin reorganization show the same phenotype (Table S1). This significant difference (p = 0.015, odds ratio of 6.5, Fisher's Exact Test) implies that hepatocyte actin dynamics is positively associated with parasite elimination throughout infection and that it is 6.5 times more likely that a parasite disappears associated with an actin event than in the absence of that event. The observation that not all actin reorganization events were associated with parasite elimination, might be due to the fact that some parasites are more fit than others and therefore offer more resistance to intense actin polymerization and consequent mechanical elimination. However, strong accumulation of actin as the one shown in Fig. 6/movie S6 was never observed around parasites that did not disappear. Importantly, the disappearance of parasites as shown here, appeared not to be a consequence of photobleaching. We observed photobleaching in several cases (Table S2; Fig. S3) and invariably found that it occurred gradually, usually in the end of the time lapse experiments and in the absence of vacuole closure after GFP bleaching. In contrast, elimination, as scored in the above numbers, could occur at any time point of the experiment and be associated with vacuole closure after GFP disappearance (Fig. S3; see Material and Methods). This clearly begs the question of where the parasite goes. One possibility would be that the parasites are “expelled” into the extracellular space as has recently been shown for cryptococci [20]. Clearly, new assays will be needed to dissect this process further.

Taken together, this data supports the hypothesis that forces generated by actin polymerization can cause parasite deformation and disappearance. Thus, host cell actin polymerization occurring around the developing parasite might constitute a mechanism through which the infected cell confines and/or eliminates the parasite mechanically. In fact, mechanisms of host cell actin-dependent extrusion are used, for instance, by *Chlamydia* to exit the host cell [21] or by *Cryptococcus* in its “lateral transfer” from an infected to a non infected macrophage [22]. On the other hand, actin reorganization may not *per se* be the most relevant mechanism in the elimination of the parasite, it may be part of a broader defence strategy to fight *Plasmodium* and that may include endosomal vesicles like lysosomes or even autophagolysosomes. For instance, in phagosomes containing mycobacteria, actin reorganization contributes to the fusion of late endocytic organelles and thus phagosome maturation and bacteria killing [17]. Although the parasitophorous vacuole is not a phagosome, the cell may attempt to destroy the foreign vacuole that starts developing inside its cytosol, by fusing it with lysosomes or by autophagy for example. Indeed actin has been shown to play a partial role in autophagy during *Shigella* infection, as the bacteria targeted for autophagy are entrapped in septin cage-like structures in an actin dependent way [23]. Actin has also been implicated in the defence against pathogens in other biological systems, such as plants that use the actin cytoskeleton against fungal penetration and phytopathogenic bacterial infection [24], [25]. Interestingly, an actin-rich structure has been also observed around *Plasmodium* ookinetes in the *Anopheles* mosquito midgut and proposed to act as a defence reaction from the vector against the invading parasite [26]. Whether the main biological relevance of hepatocyte actin reorganization around *Plasmodium* in both situations is the elimination of the parasite or whether parasite elimination occurs as a side effect of the process, remains to be established. An additional hypothesis is that actin polymerization might also be a mechanism used by the cell to remove a dead or dying parasite. Testing these hypotheses will necessitate investments into new assays, such as those recently established for the study of the vacuolar rupture after *Shigella* invasion of fibroblasts [27] as well as automated high throughput microscopy that allows following quasi-simultaneously several distantly located developing parasites in parallel. As such microscopes become available [28] a further dissection of the molecular events governing actin cloud formation and its effect on parasite disappearance can be attempted.

In conclusion, our work places developing *Plasmodium* in the group of pathogens associated with host actin dynamics. By linking the appearance of dynamic actin accumulation with the disappearance of parasites, we hypothesize that the liver cells cytoskeleton may contribute to the defence against *Plasmodium*.

## Materials and Methods

### Parasites and mice

GFP-Pb ANKA (parasite line 259cl2) sporozoites [29] were obtained from the dissection of infected female Anopheles stephensi mosquito salivary glands, produced at the IMM insectary. GFP-expressing *Toxoplasma gondii* (generously provided by *M. Meissner*, Glasgow University, UK) tachyzoites were cultivated in Vero cells that were maintained in Dulbecco's modified Eagle's medium (DMEM), supplemented with 10% foetal calf serum (FCS), 1% glutamine (Gibco/Invitrogen) and 20 µg/ml gentamycin (Gibco/Invitrogen). BALB/c mice, purchased at Instituto Gulbenkian de Ciência, were housed at Instituto de Medicina Molecular (IMM) animal house facility. All experimental protocols were performed according to EU regulations and were approved by the Instituto de Medicina Molecular Animal Care and Ethical Committee (AEC_2010_024_MM_RDT_General_IMM).

### Stable cell lines

Huh7 cells (1×10^7^) were electroporated (Gene Pulser II, Bio Rad) with 30 µg of the linearized plasmid containing the fusion construct of mCherry::human β-actin (pEGFP-C1) or mCherry::human α-tubulin (pEYFP), generously provided by F. Gertler (MIT, USA) and D. Henrique (IMM, Portugal), respectively. The selection with geneticin, G418 (0.8 mg/ml), was initiated the next day. Medium containing G418 was replaced daily for 6 days, eliminating all the non-transfected cells. Transfected cells were amplified and then sorted in a MoFlo High-Speed Cell Sorter (Beckman Coulter), using a Melles Griot 568 nm laser and a BP 630/30 filter to detect mCherry. Two populations of cells were separated, one with an intermediate fluorescent signal and another with a strong signal. The population with the stronger fluorescent signal was used throughout this work.

### Time lapse experiments and image analysis

Cells were seeded on glass bottom culture dishes (MatTek Corporation) and infected with 1×10^5^ GFP-Pb sporozoites. Infected cells were analysed by microscopy at different time points after infection. Time lapse experiments were performed on a Zeiss Axiovert 200 M inverted widefield fluorescent microscope, with a motorized stage, and equipped with a CoolSNAP HQ charge-coupled device (Roper Scientific Photometrics, Tucson, AZ) and Metamorph 6.1 software (Molecular Devices, Downingtown, PA). GFP and mCherry fluorophores were detected with the following filter sets: excitation BP450–490 nm, emission LP515; excitation 540–552 nm, emission LP590, respectively. Images were acquired with a PlanApochromat 63×/1.4 objective, at 20 s time intervals. *T. gondii* and beads (Polybead Amino 3 Micron Microspheres, Polyscience, Inc.) experiments were performed in conditions as similar as possible to those used for *P. berghei*: (i) dissection products of non infected salivary glands were added to cells that had internalized beads and *T. gondii* infected cells; (ii) time lapse experiments were performed between 10 and 16 hours post internalization or infection, corresponding to the interval of *P. berghei* infection where there is the highest percentage of parasites associated with host actin reorganization and (iii) microscope settings and acquisition intervals were the same as for *Plasmodium* time lapse experiments.

Regarding parasite elimination, only movies showing vacuole closure after parasite disappearance were considered in the analysis. GFP parasites that disappeared without vacuole closure were not considered (Fig. S3B). We attributed those events to photobleaching as they invariably occurred at the end of a time-lapse series. Six out of 485 parasites not associated with host actin reorganization disappeared without vacuole closure and 2 out of 77 parasites, associated with host actin reorganization also disappeared without the vacuole closing (p = 0.3, Fisher's Exact Test). Examples of parasites not considered in the analysis can be observed in Fig. S3B (Table S2). During all time lapse experiments, cells were kept at 37°C and 5% CO_2_. Image files were processed using ImageJ 1.38 h software.

### Immunofluorescence

Cells were plated on 12 mm glass coverslips and fixed in 4% paraformaldehyde, for 10 minutes. After fixation, they were permeabilized in 0,1% Triton X-100 (Calbiochem) and blocked with 10% bovine serum albumin (BSA) before incubation for 1 hour with the respective antibodies or phalloidin (Molecular Probes/Invitrogen) diluted in blocking solution. Nuclei were stained with 4′,6-diamidino-2-phenylindole (DAPI). Images were obtained on a spinning disc laser confocal microscope (Revolution System, Andor Technology, Belfast, UK) or on a Zeiss LSM 510 META (Zeiss, Oberkochen, Germany).

### Immunohistochemistry of mouse liver slices

BALB/c was infected by intra-venous (i.v.) inoculation with GFP-Pb sporozoites. For phalloidin labeling, mice were infected with 500 000 GFP-Pb sporozoites. Livers were perfused with PBS and harvested 24 hours after sporozoite injection. Tissues were then fixed in 4% PFA for 24 hours at 4°C, washed with PBS for 1 hour and then sliced into 50 µm sections using a vibratome (VT1000S, Leica). Sections were again fixed for 5 minutes with 4% PFA, permeabilized for 1 hour with 0.3% Triton X-100 and blocked for 2 hours, with 1% BSA. Sections were then incubated for 24 hours at 4°C in blocking solution containing rabbit anti-GFP FITC (Molecular Probes/Invitrogen) and AlexaFluor 594 phalloidin (Molecular Probes/Invitrogen). Nuclei were stained for 30 minutes with DAPI. Images were acquired in a Zeiss LSM 510 META (Zeiss, Oberkochen, Germany).

### *P. berghei*-infected hepatoma cell microarray analysis

The GeneChip® Mouse Expression 430 2.0 array contains45000 probesets, covering 39000 transcripts and variants from over 34000 well characterized mouse genes. Data was obtained from previous publication [18] and all raw data is MIAME compliant and accessible through Array Express or GEO, accession number: E-MEXP-667.

### Lentiviral shRNA knockdown of Gelsolin

Plasmids encoding lentiviruses expressing shRNAs were obtained from the library of the RNAi Consortium (TRC) [30]. Viruses were produced as previously described [30]. Five different hairpins were initially used and their GSN knockdown efficiency was compared by qRT-PCR against a scramble hairpin. All shRNA sequences efficiently knockdown GSN (>80%) and the following shRNA sequence,CCGGCGACAGCTACATCATTCTGTACTCGAGTACAGAATGATGTAGCTGTCGTTTTT, was chosen to use in the live imaging experiments. For lentivirus infection, 5×10^3^ Huh7 mCherry::β-actin cells were seeded on a 96-well plate. In the following day, 10 µl of virus were added to each well, in the presence of medium containing 8 µg/ml of polybrene (Sigma) and the plate was centrifuged at 974 g for 90 minutes, at 37°C. The medium was then removed and supplemented RPMI was added. Selection of non-transduced cells started 48 hours later, with 4 µg/ml of puromycin (Calbiochem). Cells were infected and used in time lapse experiments after 48 hours of selection, GSN expression was quantified by qRT-PCR and Western Blot as described before [31]. Antibodies used in the Western blot include anti-GSN (BD Transduction Laboratories), anti-α tubulin (Sigma) and HRP conjugated anti-mouse (Amersham).

### Statistical Analysis

Several data are presented in the form of percentages. For low percentages (<8%), we computed exact confidence intervals for the respective probability. For the remaining percentages, we calculated traditional asymptotic confidence intervals based on the standard error (s.e.) associated with the estimates. Pearson independence test for two-way contingency tables was used to (i) assess culture-plate effects in time lapse experiments performed on different days and (ii) compare the frequency of parasites or beads associated with host actin reorganization. In (i), the null hypothesis was that all different plates referring to the same infection period show a similar relative frequency of parasites or beads associated with host actin reorganization. Since this hypothesis could be accepted at the 5% significance level, data from different plates imaged at different days were pooled together for further analysis. In (ii), the null hypothesis is that there is no difference between the groups. Fisher's exact test for 2×2 tables was used to assess whether the disappearance of parasites was or not associated with host actin reorganization. The application of this exact test is justified by the presence of an unbalanced 2×2 table, which would lead to unreliable p-values for the traditional Pearson's independence test. Since we could reject the independence hypothesis between parasite disappearance and host actin reorganization, we extended further the analysis by calculating the odds ratio and the respective 95% confidence interval. Student's T test was used to analyze the data from the remaining experiments because the data was following a Gaussian distribution. In all above-mentioned tests, the null hypothesis was accepted when the p-value>0.05 or rejected, otherwise. In the same vein, all confidence intervals were computed at a 95% confidence level. All statistical data analysis was carried out using SPSS 11.0. for windows and R software (http://www.r-project.org).

## Supporting Information

Figure S1 **Huh7 cells stably expressing mCherry::human β-actin or mCherry::human α-tubulin.** Immunofluorescence of Huh7 mCherry::human β-actin and Huh7 mCherry::human α-tubulin stained with phalloidin or an antibody anti-α-tubulin respectively (red: mCherry::β-actin or mCherry::α-tubulin; green: phalloidin Alexa Fluor 488 or anti-α-tubulin antibody; blue: nuclei), scale bars represent 10 µm.
(TIF)

Figure S2 **Comparison of** ***P.berghei*** **infection in Huh7 cells vs Huh7 mCherry:: human β-actin or Huh7 mCherry::human α-tubulin cell lines, 48 hours after infection.** Cells were infected with 3×10^4^ GFP-Pb sporozoites and infection was measured by flow cytometry.
(TIF)

Figure S3 ***P. berghei*** **elimination** ***versus*** **photobleaching during time lapse experiments.** (**A**) GFP-Pb elimination in the absence of host actin reorganization. Note that the left parasite disappears completely, while the right parasite remains. (**B**) GFP-Pb photobleaching. Both parasites gradually lose their fluorescence during the time lapse experiment. The place where parasites were visible remains in the mCherry::β-actin channel. Arrows indicate the position of where the parasite is after bleaching. (red: mCherry::β-actin; green: GFP-Pb; grey pictures represent single channels); Scale bars represent 10 µm.
(TIF)

Movie S1 Time lapse experiment showing no tubulin reorganization around GFP-Pb, representative of the 238 parasites analysed with this cell line (red: mCherry::α-tubulin; green: GFP-Pb; grey pictures represent single channels), scale bar represents 10 µm.
(MOV)

Movie S2 Time lapse experiment of Huh7 mCherry::β-actin infected with GFP-Pb, showing a transient and symmetrical reorganization of host actin around the parasite (red and grey: mCherry::β-actin; green: GFP-Pb), scale bar represents 10 µm.
(MOV)

Movie S3 Comparative actin reorganization events in Huh7 mCherry::β-actin infected with *P. berghei* (left), *T. gondii* (middle) and 3 µm beads (right), respectively. Only mCherry::β- actin channels are shown. Squares in the initial frames represent the regions where the parasites or the beads are located. The permanent fluorescent ring around the bead corresponds to autofluorescence, as it is also observed in beads imaged in the absence of cells, when the same microscope settings are employed, scale bars represent 10 µm.
(MOV)

Movie S4 Asymmetrical actin clouds around GFP-Pb (red and grey: mCherry::β-actin; green: GFP-Pb), scale bar represents 10 µm.
(MOV)

Movie S5 Tail-like actin cloud associated with GFP-Pb (red and grey: mCherry::β-actin; green: GFP-Pb), scale bar represents 10 µm.
(MOV)

Movie S6 Host actin reorganization event coinciding with *P. berghei* deformation and disappearance (red: mCherry::β-actin; green and grey: GFP-Pb), scale bar represents 10 µm.
(MOV)

Table S1 GFP-Pb elimination in the presence and absence of actin reorganization.
(DOCX)

Table S2 GFP-Pb photobleaching in the presence and absence of host actin reorganization.
(DOCX)
