# Supplementary figures and images for: Highly Dynamic Host Actin Reorganization around Developing Plasmodium Inside Hepatocytes
Source: PLoS One. 2012 Jan 6;7(1):e29408. doi: 10.1371/journal.pone.0029408 (PMC3253080; doi:10.1371/journal.pone.0029408)

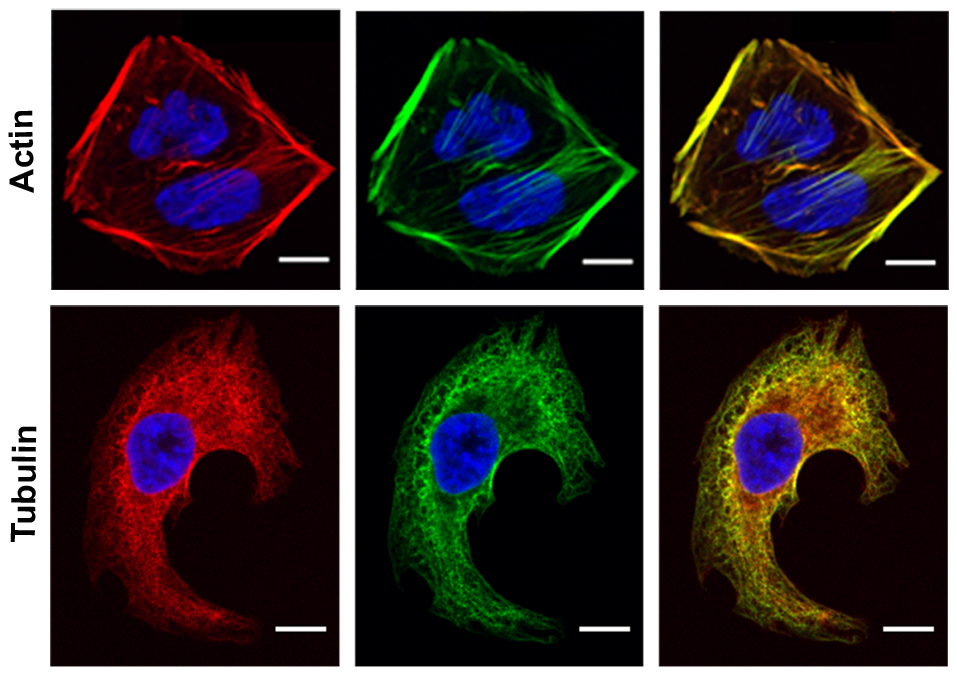

Supplement: Figure S1 — Huh7 cells stably expressing mCherry::human β-actin or mCherry::human α-tubulin. Immunofluorescence of Huh7 mCherry::human β-actin and Huh7 mCherry::human α-tubulin stained with phalloidin or an antibody anti-α-tubulin respectively (red: mCherry::β-actin or mCherry::α-tubulin; green: phalloidin Alexa Fluor 488 or anti-α-tubulin antibody; blue: nuclei), scale bars represent 10 µm. (TIF) [file pone.0029408.s001.tif]

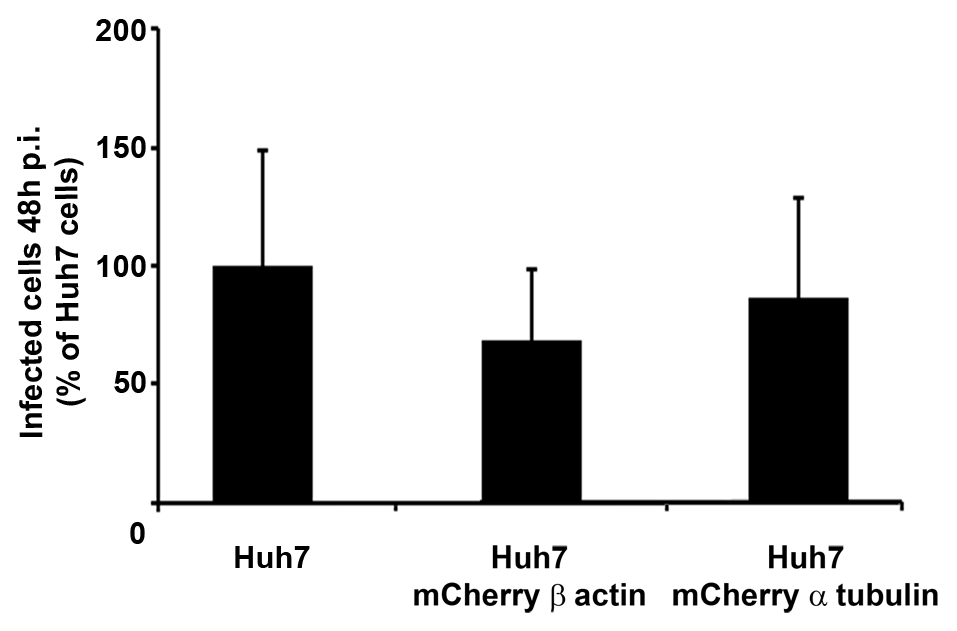

Supplement: Figure S2 — Comparison of P.berghei infection in Huh7 cells vs Huh7 mCherry:: human β-actin or Huh7 mCherry::human α-tubulin cell lines, 48 hours after infection. Cells were infected with 3×10^4^ GFP-Pb sporozoites and infection was measured by flow cytometry. (TIF) [file pone.0029408.s002.tif]

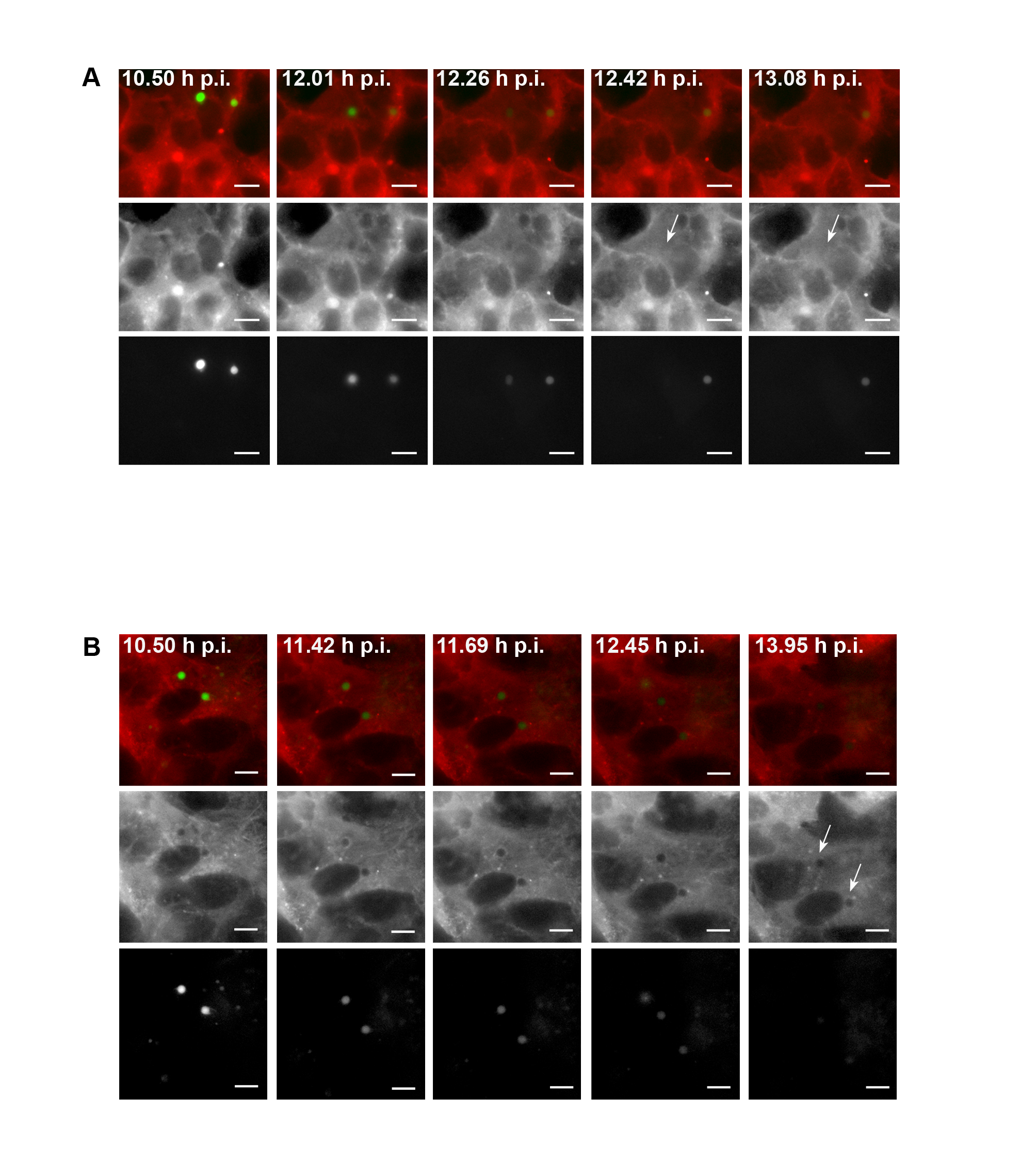

Supplement: Figure S3 — P. berghei elimination versus photobleaching during time lapse experiments. (A) GFP-Pb elimination in the absence of host actin reorganization. Note that the left parasite disappears completely, while the right parasite remains. (B) GFP-Pb photobleaching. Both parasites gradually lose their fluorescence during the time lapse experiment. The place where parasites were visible remains in the mCherry::β-actin channel. Arrows indicate the position of where the parasite is after bleaching. (red: mCherry::β-actin; green: GFP-Pb; grey pictures represent single channels); Scale bars represent 10 µm. (TIF) [file pone.0029408.s003.tif]
