# Supplementary material for: Highly Dynamic Host Actin Reorganization around Developing Plasmodium Inside Hepatocytes
Source: PLoS One. 2012 Jan 6;7(1):e29408. doi: 10.1371/journal.pone.0029408 (PMC3253080; doi:10.1371/journal.pone.0029408)
Supplement: Table S1 — GFP-Pb elimination in the presence and absence of actin reorganization. (DOCX) [file pone.0029408.s010.docx]

**Supporting Tables**

**Table S1-** GFP-Pb elimination in the presence and absence of actin reorganization

|  | **DISAPPEARANCE** | **NO DISAPPEARANCE** | **TOTAL** |
| --- | --- | --- | --- |
| **ACTIN REORGANIZATION** | 4 | 73 | 77 |
| **NO ACTIN REORGANIZATION** | 4 | 481 | 485 |
| **TOTAL** | 8 | 554 | 562 |

(p=0.015, Fisher´s Exact Test)
