# Supplementary material for: Highly Dynamic Host Actin Reorganization around Developing Plasmodium Inside Hepatocytes
Source: PLoS One. 2012 Jan 6;7(1):e29408. doi: 10.1371/journal.pone.0029408 (PMC3253080; doi:10.1371/journal.pone.0029408)
Supplement: Table S2 — GFP-Pb photobleaching in the presence and absence of host actin reorganization. (DOCX) [file pone.0029408.s011.docx]

**Table S2-** GFP-Pb photobleaching in the presence and absence of host actin reorganization

(p=0.3, Fisher´s Exact Test)

|  | **GFP PHOTOBLEACH** | **NO GFP PHOTOBLEACH** | **TOTAL** |
| --- | --- | --- | --- |
| **ACTIN REORGANIZATION** | 2 | 75 | 77 |
| **NO ACTIN REORGANIZATION** | 6 | 479 | 485 |
| **TOTAL** | 8 | 554 | 562 |
